# Specific Proton-Donor Properties of Glycine Betaine. Metric Parameters and Enthalpy of Noncovalent Interactions in its Dimer, Water Complexes and Crystalline Hydrate

**DOI:** 10.3390/ijms241612971

**Published:** 2023-08-19

**Authors:** Nikita E. Frolov, Anastasia V. Shishkina, Mikhail V. Vener

**Affiliations:** 1 V. M. Gorbatov Federal Research Center for Food Systems, Talalikhina St., 26, Moscow 109316, Russia; frolovne24@gmail.com; 2 Department of Physics and Engineering Environmental Protection, Northern (Arctic) Federal University, Severnaya Dvina Emb. 17, Arkhangelsk 163001, Russia; a.shishkina@narfu.ru; 3 Kurnakov Institute of General and Inorganic Chemistry, Russian Academy of Sciences, Leninskii prosp. 31, Moscow 119991, Russia

**Keywords:** trimethylglycine, intra- and intermolecular interactions, intermolecular H-bonds, C–H stretching vibrations, ^1^H chemical shifts

## Abstract

Trimethylglycine (glycine betaine, GB) is an important organic osmolyte that accumulates in various plant species in response to environmental stresses and has significant potential as a bioactive agent with low environmental impact. It is assumed that the hydration of GB is playing an important role in the protective mechanism. The hydration and aggregation properties of GB have not yet been studied in detail at the atomistic level. In this work, noncovalent interactions in the GB dimer and its complexes with water and crystalline monohydrate are studied. Depending on the object, periodic and non-periodic DFT calculations are used. Particular attention is paid to the metric parameters and enthalpies of intermolecular hydrogen bonds. The identification of noncovalent interactions is carried out by means of the Bader analysis of periodic or non-periodic electron density. The enthalpy of hydrogen bonds is estimated using the Rosenberg formula (PCCP 2 (2000) 2699). The specific proton donor properties of glycine betaine are due to its ability to form intermolecular C–H∙∙∙O bonds with the oxygen atom of a water molecule or the carboxylate group of a neighboring GB. The enthalpy of these bonds can be significantly greater than 10 kJ/mol. The water molecule that forms a hydrogen bond with the carboxylate group of GB also interacts with its CH groups through lone pairs of electrons. The C–H∙∙∙O bonds contribute up to 40% of the total entropy of the GB–water interaction, which is about 45 kJ/mol. The possibility of identifying C–H∙∙∙O bonds by the proton nuclear magnetic resonance method is discussed.

## 1. Introduction

In recent years, much attention has been paid to the study of the interaction between trimethylglycine (glycine betaine, GB) and water in order to understand the structure–performance relationships for chemical, biological and pharmaceutical applications [1,2]. Experimental and theoretical investigations [3,4,5,6,7,8,9,10] have provided valuable insights into the solvent effects on the zwitterionic GB. In contrast to the solvation of aliphatic amino acid zwitterions [11,12,13,14], where the hydration shells of the amino group (NH_3_^+^), carboxylate group (COO^−^) and methylene group (CH_2_) should be considered [15,16], it is necessary to focus on the interaction of COO^−^ with water in GB. Such interaction has been studied in detail for carboxylate ions [17,18], subunits of photosystem II [19], anion of diclofenac [20] and anions of some drug compounds [21]. In these cases, the main attention has been paid to the description of intermolecular hydrogen bonds (H-bonds) formed by the COO^−^ group of a solute with water molecules. However, GB has several significant differences from the above compounds. First, it exists as a zwitterion not only in condensed media, but also in vacuum. Second, the presence of a methylene group in the GB zwitterion indicates the presence of acidic protons capable of forming intermolecular C–H∙∙∙O bonds. The latter play an important role in biologically active systems [22,23] and multicomponent drug crystals [24,25]. As a result, GB can be treated uniformly in vacuum and in the condensed phase. (Proton transfer should be considered for amino acids in vacuum. This complicates the theoretical treatment and often leads to the use of different calculation levels for amino acids in vacuum and in crystals [26]). In addition, the structure of its hydration shell may differ from that of aliphatic amino acids, since GB can form C–H∙∙∙O_w_ bonds [10], where “w” denotes the oxygen atom of the water molecule.

This study assumes that a certain noncovalent intermolecular interaction is due to the existence of a bond path (i.e., a bond critical point) [27] between a pair of atoms belonging to different molecules. The identification of noncovalent interactions is based on the Bader analysis of the electron density obtained for the optimized non-periodic or crystalline structures using density functional theory (DFT). The enthalpy of intermolecular H-bonds is estimated using the Rosenberg approach [28], and the energy of intermolecular H∙∙∙H-bonds is evaluated using the properties of electron density at the bond critical point [29]. The applicability of these approaches to estimating the enthalpy/energy of intermolecular H-bonds is given elsewhere [30]. A detailed study of the intramolecular H-bonds [10,31] is beyond the scope of this study.

We will focus on the geometry and enthalpy of intermolecular H-bonds in the GB water complexes, its dimer and crystalline monohydrate. At the first stage, the microsolvation of the GB zwitterion is considered and the obtained configurations of hydration shells with different numbers of water molecules are compared with those in amino acid zwitterions and carboxylate ions. At the second stage, the structure of the GB dimer is calculated and the enthalpy of noncovalent interactions in it is estimated. At the third stage, the enthalpy of noncovalent interactions in GB crystalline monohydrate is evaluated. The obtained results allowed us to reveal the specific proton-donor properties of GB compared to amino acid zwitterions or carboxylate ions, and led to a valuable understanding of the interaction between GB and water. It should be emphasized that the same theoretical level was used for non-periodic and periodic DFT calculations.

## 2. Results

### 2.1. Metric Parameters and Enthalpy of Intermolecular H-Bonds in Complexes of GB with Water

The paper considers GB complexes with water, in which water molecules form O_w_–H∙∙∙O bonds with COO^−^ and C–H∙∙∙O_w_ bonds with the C–H of the methylene and methyl groups. Sequential hydration, i.e., the successive addition of water molecules to the respective zwitterion or ion, is usually studied [12,18,32,33]. The number of water molecules forming the first solvation shell of GB, i.e., interacting with GB through H-bonds, is about 7 [34]. In this case, up to five water molecules interact directly with COO^−^ [18,35]. The energy of structures in which the water molecules do not interact directly with the COO^−^ group (Figure 8 in [18]) is usually much higher than the energy of the global minimum structure (>>*k*_B_*T*, where *k*_B_ is the Boltzmann constant and *T* = 300 K). The localization of such structures is beyond the scope of this work. For “incomplete” hydration shells, in addition to the structure of the global minimum, there are several possible structures corresponding to local minima whose energy is quite close to that of the global minimum. The energies of the structures of the low-lying local and global minima may be very close, ~ *k*_B_*T*, and their relative stability can vary depending on the calculation level [32]. In this work, the main attention is paid to the type, number, metric parameters and enthalpies of intermolecular H-bonds in the structures of the global minimum and several low-lying local minima of GB•(H_2_O)_n_ complexes, where n = 1, 2, …5. The calculations were performed in the B3LYP/6-311++G**, wB97XD/aug-cc-pVDZ and B3LYP/6-31G** approximations, since the latter approximation was used in the calculations of the GB crystalline hydrate. An exact determination of the relative energies of the local minima structures is not within this research focus. 

**Figure 1:**
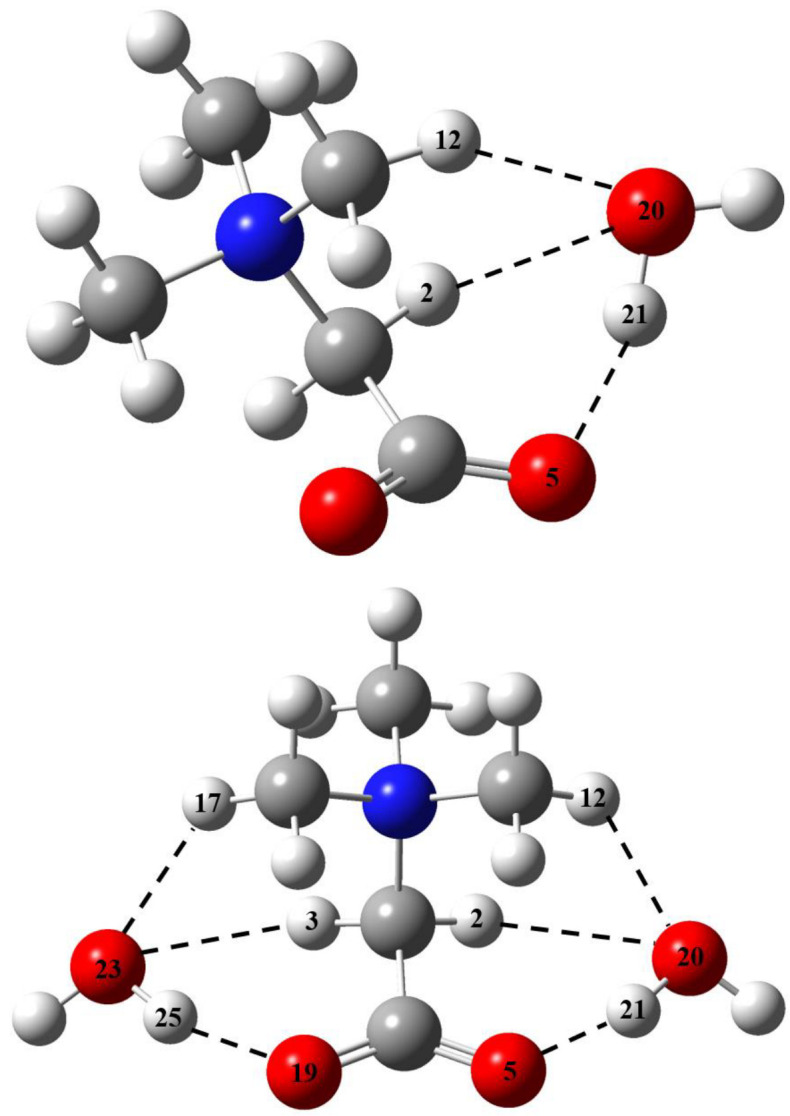
The global minimum structures of GB•(H_2_O) (**upper panel**) and GB•(H_2_O)_2_ (**lower panel**). H-bonds are indicated by dotted lines.

For simplicity, the term “global minimum structure” will be used below to designate the structure of the GB•(H_2_O)_n_ complex found in the B3LYP/6-31G** approximation. Such a structure of the GB•(H_2_O) complex is shown in Figure 1 (upper panel). The water bridges the carboxylate group and the C–H of the methylene and methyl groups. It forms the O_w_–H∙∙∙O bond of moderate strength [36] ~27 kJ/mol as a proton donor and two C–H∙∙∙O_w_ bonds as proton acceptors. The total enthalpy of the latter is ~23 kJ/mol (Table S1). 

The considered structure of GB•(H_2_O) is similar to the 1Z-a structure of the glycine zwitterion with water [32]. However, this is inconsistent with the structures of global minima found for the acetate ion [18,33] and for the GB•(H_2_O) complex [10]. A possible reason for this difference is related to the fact that C–H∙∙∙O_w_ bonds were not considered or were missing in [10,18,33]. The enthalpies of intermolecular H-bonds in the low-lying local minima structures of GB•(H_2_O) are given in Table S2. All structures have one O_w_–H∙∙∙O bond and two C–H∙∙∙O_w_ bonds(Figure S1). In the B3LYP/6-311++G** and wB97XD/aug-cc-pVDZ approximations, all three structures are characterized by very close stability.

Figure 1 (lower panel) shows the global minimum structure of the GB•(H_2_O)_2_ complex. It is “unique” because it is not realized in partially hydrated amino acid zwitterions [12,32] and acetate ions [18,33]. The total enthalpy of two O_w_–H∙∙∙O bonds is ~55 kJ/mol, and the total enthalpy of four C–H∙∙∙O_w_ bonds is about 45 kJ/mol (Table S1). The structures corresponding to the local minima are shown in Figure S2, and the enthalpies of the intermolecular H-bonds occurring in them are listed in Table S3.

**Figure 2:**
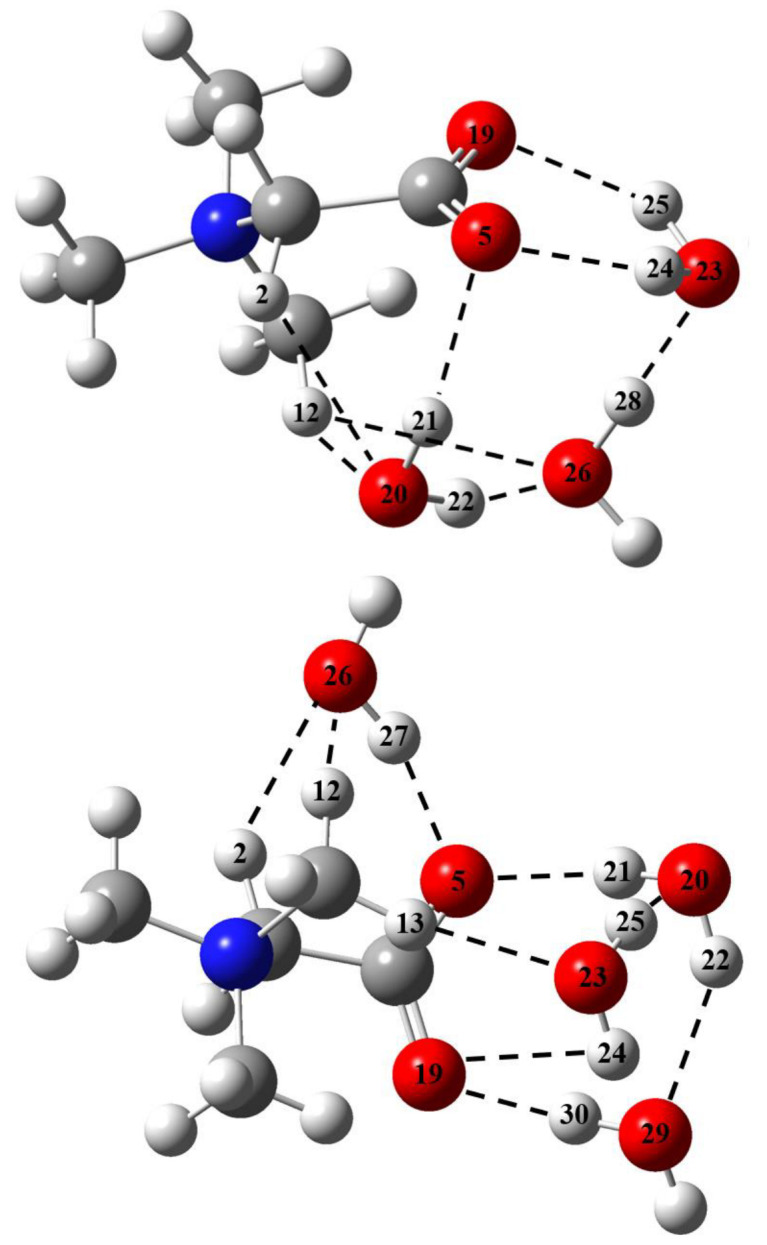
The global minimum structures of GB•(H_2_O)_3_ (**upper panel**) and GB•(H_2_O)_4_ (**lower panel**). H-bonds are indicated by dotted lines.

In Figure 2 (upper panel), the structure of the global minimum GB•(H_2_O)_3_ is shown. It resembles microsolvated structures of GB with three water molecules (see Figure 3a in Ref. [9]). The total enthalpy of four O_w_–H∙∙∙O bonds is ~100 kJ/mol, and the total enthalpy of two C–H∙∙∙O_w_ bonds is less than 30 kJ/mol (Table S1). A feature of this structure is the presence of a water molecule that does not form H-bonds with GB. The structures corresponding to the low-lying local minima structures are shown in Figure S3, and the enthalpies of the intermolecular H-bonds occurring in these structures are listed in Table S4. 

**Figure 3:**
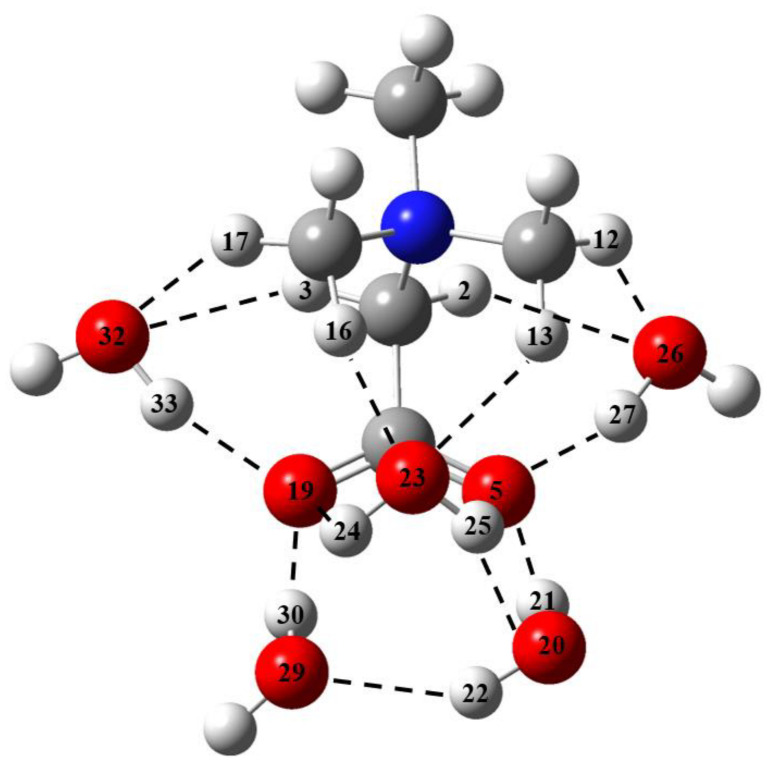
The global minimum structure of GB•(H_2_O)_5_. H-bonds are indicated by dotted lines.

The global minimum structures of GB•(H_2_O)_4_ and GB•(H_2_O)_5_ are shown in Figure 2 (lower panel) and Figure 3, respectively. All the water molecules in them form H-bonds with the COO^−^ group. According to Tables S1 and S5, the number of C–H∙∙∙O_w_ bonds in the structures of the considered complexes may be comparable to the number of O_w_–H∙∙∙O bonds.

The results obtained allow us to draw the following conclusions. The B3LYP/6-31G** approximation gives the metric parameters and enthalpies of the O_w_–H∙∙∙O bonds very close to the values calculated in the B3LYP/6-311++G** approximation. At the same time, B3LYP/6-31G** systematically overestimates the metric parameters and enthalpies of the C–H∙∙∙O_w_ bonds (~2 kJ/mol) compared to B3LYP/6-311++G**. The results of the wB97XD/aug-cc-pVDZ calculations lie between the values obtained by B3LYP with modest and extended basis sets. The enthalpies of the H-bonds found using Equation (1) are in reasonable agreement with the energies estimated from Equation (2). All this points to the reliability of the obtained results.

**Scheme 1:**
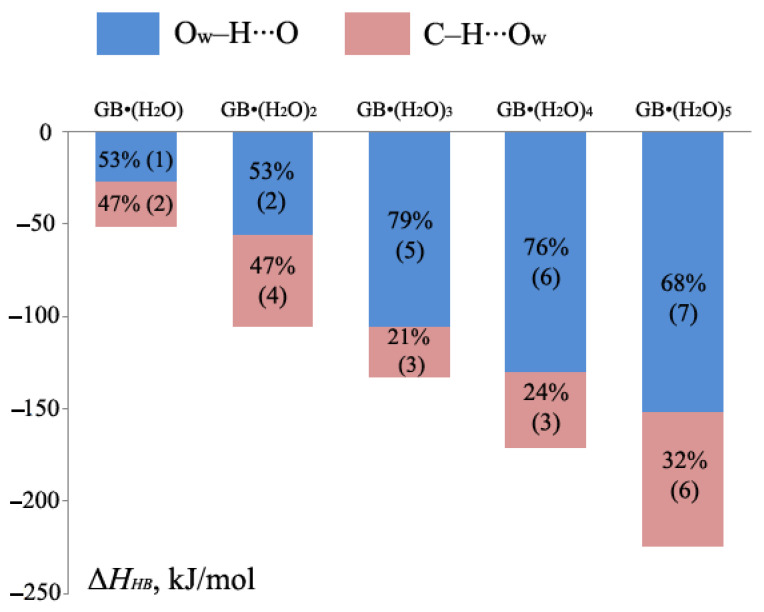
Total enthalpy of intermolecular H-bonds (Δ*H*_HB_) in the global minimum structures of the GB•(H_2_O)_n_ complexes, n = 1, 2,…5. The relative contribution of O_w_–H∙∙∙O and C–H∙∙∙O_w_ bonds to the total enthalpy is given as a percentage. The number of H-bonds of the corresponding type is given in parentheses.

Scheme 1 reflects the following specific features of GB complexes with water molecules. In structures with n = 1 and 2, O_w_–H∙∙∙O bonds as well as C–H∙∙∙O_w_ bonds contribute equally to the total energy of the complexes. In complexes with n = 3, 4 and 5, the proportion of C–H∙∙∙O_w_ bonds increases with the number of water molecules and reaches 32% in the case of the GB•(H_2_O)_5_ structure. From Scheme 1, it can be seen that O_w_–H∙∙O bonds account on average for ~60% of the total energy of intermolecular interactions in the considered complex. About 40% of the energy is associated with the formation of C–H∙∙∙O_w_ bonds.

Similar to crystalline hydrates [37,38], the water molecule can form three H-bonds with gGB: either two O_w_–H∙∙∙O bonds as a proton donor and one C–H∙∙∙O_w_ bond as a proton acceptor, or vice versa with a total enthalpy of about 45 kJ/mol. As a result, the total enthalpy of intermolecular H-bonds in the GB•(H_2_O)_5_ complex is greater than 200 kJ/mol. 

### 2.2. GB Dimer in Vacuum

Intermolecular interactions of organic molecules and ions in gas [39,40,41,42,43] and condensed phases [44,45,46,47,48] have been studied experimentally and theoretically during the last two decades. The quantitative description of the intermolecular interaction in the GB dimer deserves special attention for the following reason. The interaction of two zwitterions in vacuum allows us to compare the interaction energy obtained by two approaches: total energies and Bader’s approach [27]. In the latter case, several empirical approaches have been proposed to estimate the energy of noncovalent interactions [49,50]. 

**Figure 4:**
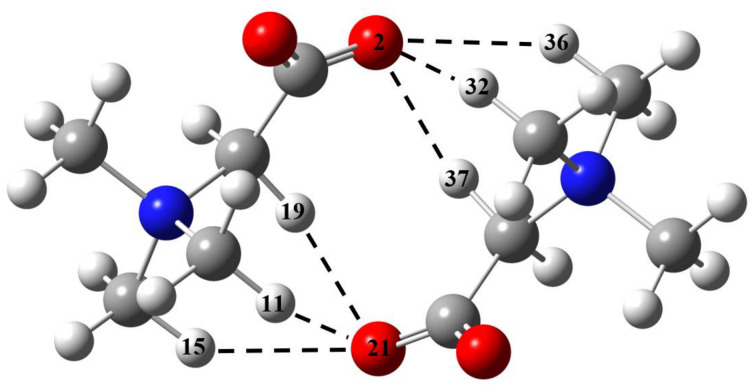
GB dimer. C–H∙∙∙O bonds are indicated by dotted lines.

The mutual orientation of the molecules in the GB dimer is shown in Figure 4. From the data given in Table 1, it can be concluded that the GB dimer is stabilized by a large number of intermolecular C–H∙∙∙O bonds. These bonds are formed by the hydrogens of the methylene and methyl groups of one GB molecule with the oxygen of the carbonyl group of another GB molecule, with enthalpies ranging from ~10 to ~14 kJ/mol. The total enthalpy of these bonds is about 75 kJ/mol (B3LYP/6-31G**) and 71.5 kJ/mol (wB97XD/aug-cc-pVDZ).

The interaction energy in the dimer was also calculated using the energy of the GB molecule and its dimer, taking into account the BSSE error (Section 4.1). In this case, the equilibrium geometry of the dimer obtained in the corresponding approximation was used. The value of the interaction energy in the GB dimer, calculated from the total energies taking into account the BSSE error, is 110 kJ/mol (B3LYP/6-31G**) and 145 kJ/mol (wB97XD/aug-cc-pVDZ). Thus, B3LYP/6-31G** significantly underestimates the interaction energy in the dimer compared to wB97XD/aug-cc-pVDZ. 

The different interaction energies obtained using the total energy and the Bader approach are explained by the significant dipole–dipole interaction in the dimer due to the large value of the dipole moment of the GB molecule, 10.8 D (B3LYP/6-31G**) and 12.0 D (wB97XD/aug-cc-pVDZ).

### 2.3. The GB Crystalline Monohydrate

A quantitative description of noncovalent interactions in molecular crystals is not easy. From an experimental point of view, there must be a known number of noncovalent interactions of the same type, such as in water ice [51]. Then, the enthalpy of the desired interaction can be estimated from the enthalpy of sublimation. From a computational point of view, it is necessary to identify noncovalent interactions (there are many short contacts in molecular crystals) and estimate the energy of each interaction (formulas using different electron density characteristics lead to different energy values [52]). The crystalline hydrates of organic molecules are convenient objects for studying noncovalent interactions in solids because (i) they are often formed under traditional crystallization conditions [53,54,55], and (ii) the enthalpies of intermolecular H-bonds can be obtained by a method using the metric properties of these bonds [28].

**Figure 5:**
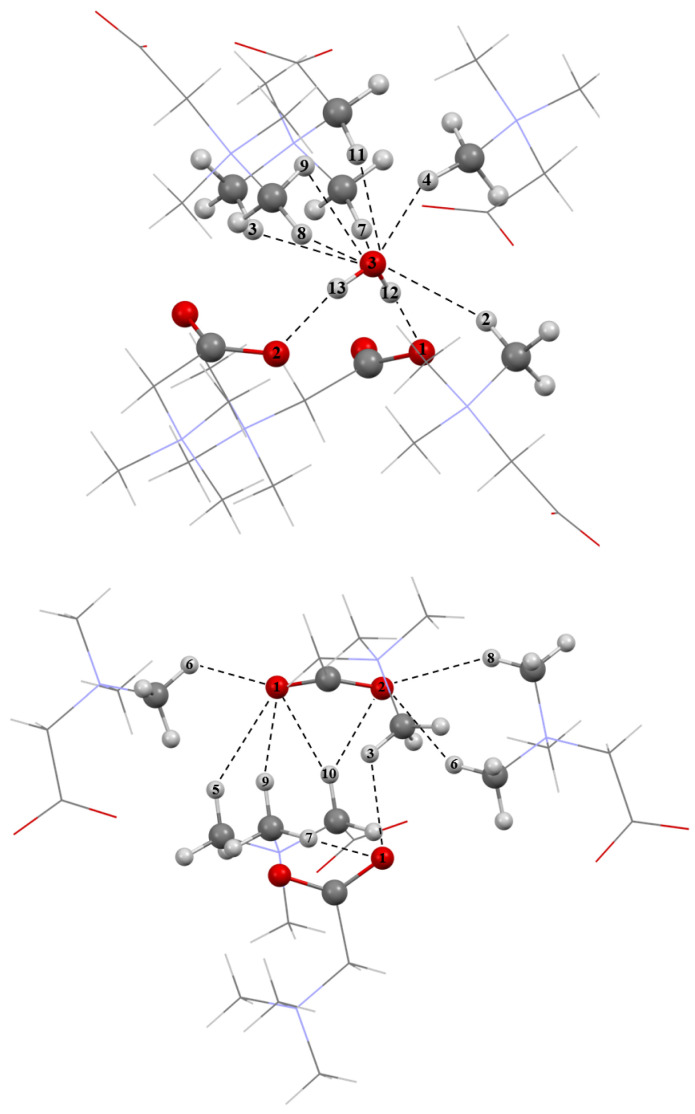
Intermolecular H-bonds in GB crystalline monohydrate. **Upper** figure: H-bonds between water and GB. **Bottom** picture: H-bonds between GB molecules.

Intermolecular interactions in the GB crystalline monohydrate can be divided into two types: water–GB and GB–GB. (Water-–water interactions are not realized in this crystal). Table 2 shows the water–betaine interaction energies. These interactions are shown in Figure 5. It follows that the B3LYP/6-31G** and PBE-D3/6-31G** approximations lead to close values of the distances H∙∙∙O and –Δ*H*_HB_ in the considered crystal; the total enthalpy of water–betaine interactions is greater than that of betaine–betaine interactions. This indicates the structure-determining role of the water molecule.

A comparison of Table S1 and Table 2 shows that:

(1) In the GB crystalline monohydrate, there is a significant strengthening of classical H-bonds in comparison with GB–water complexes in the gas phase;
(2) The number of C–H∙∙∙O_w_ bonds in the crystalline monohydrate also increases in comparison with the GB–water complexes in the gas phase.

In GB crystalline monohydrate [56], a large number of GB–GB intermolecular interactions are also realized, as seen in Table 2. A comparison of Table 1 and Table 2 shows that:

(1) In the GB crystalline monohydrate, the energy of the C–H∙∙∙O bond does not change significantly compared to the corresponding bonds in the GB dimer in the gas phase;
(2) The number of intermolecular bonds in a crystal is much larger than in a dimer. This is due to the fact that in the considered crystal, the GB and water molecules interact with several neighboring GB molecules.

From the data presented in Tables S6 and S7, it follows that H∙∙∙H interactions are also realized in the dimer and crystalline monohydrate of GB, the energy of which varies from 4 to 9 kJ/mol. The energy of such interactions can reach 20 kJ/mol [57].

## 3. Discussion

**Figure 6:**
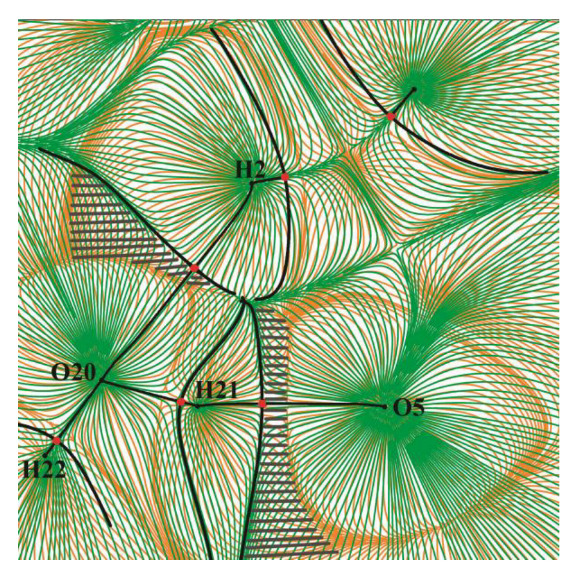
Superposition of the electrostatic potential gradient field (orange) and electron density gradient field (green) in the plane of O5-H21-H2 atoms for GB•(H_2_O) (Figure 1, upper panel). Interatomic surfaces projections are in black for ρ-basin. QTAIM *ρ*(***r***) topological bond (3, –1) critical points are drawn in red.

Interatomic surfaces in gradient fields of electron density, *ρ*, and electrostatic potential, φ, allowed us to identify the atomic *ρ* -basins and φ -basins, respectively. The first one defines chemically bonded atoms and the second one determines electrically neutral atomic fragments within a common electron-nuclear system. We remember that the electron density within each φ-basin is attracted to the corresponding nucleus. Considering the superposition of the *ρ*-basins and φ-basins of adjacent atoms A and B of a different kind, we note that the part of the electron density belonging to atom A falls into the φ-basin of atom B. Figure 6 shows that the *ρ*-basin and φ-basin overlap, forming intermolecular hydrogen bonds: O_w_–H∙∙∙O and C–H∙∙∙ O_w_ (these regions are shaded). The size of these regions not only indicates a significant electrostatic contribution to the formation of the H-bonds, but also that the acidity of the H2 atom is comparable to that of the H21 atom.

The question is whether it is possible to identify C–H∙∙∙O bonds by any experimental method. IR spectroscopy [58] and proton nuclear magnetic resonance (^1^H NMR) [59,60] seem to be the most suitable. 

**Figure 7:**
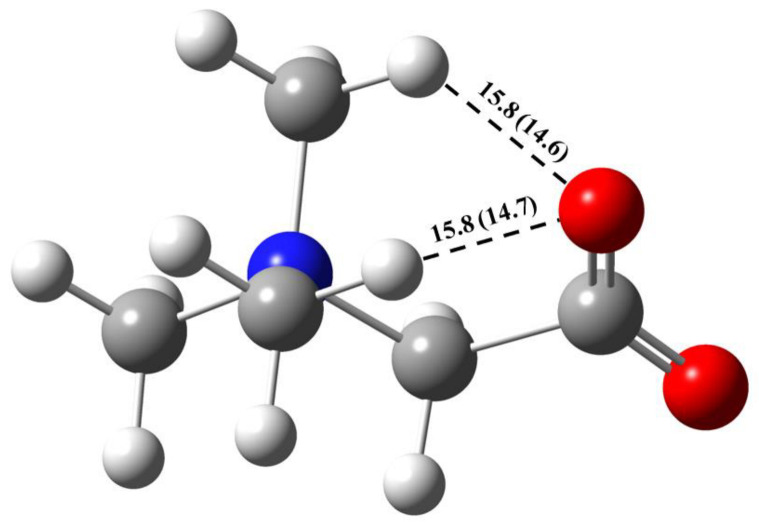
Intramolecular C–H∙∙∙O bonds in the GB molecule. Their enthalpies (B3LYP/6-31G**) are given in kJ/mol. The values calculated at the B3LYP/aug-cc-pVTZ level are given in parentheses.

The calculated frequencies of the C–H stretching vibrations of the methylene and methyl groups of GB are in the range of 3060–3180 cm^−1^. Unscaled values of the frequencies are in agreement with the available experimental data [61]. In the GB complex with five water molecules, the frequency of these vibrations is in the range of 3075–3210 wavenumbers, i.e., it is slightly shifted to the blue region in comparison with GB. The small value of the shift in the frequencies of the C–H stretching vibrations has a simple explanation. In the GB molecule, two intramolecular C–H∙∙∙O bonds are realized (Figure 7). We conclude that vibrational spectroscopy is hardly applicable for the experimental identification of C–H∙∙∙O bonds in the GB complex with water. 

The chemical shift, δ, is calculated from the absolute shielding of the nucleus in the molecule of interest, σ, and in a reference compound, σ^ref^ [62]. With a high degree of accuracy, δ ≈ σ^ref^ -σ [63]. Tetramethylsilane is well known as a reference standard for the ^1^H NMR chemical shift [64]. Σ^ref^ is 31.74 ppm for the isolated TMS molecule computed at the B3LYP/aug-cc-pVTZ level [65]. The structure and absolute shielding of ^1^H atoms in GB and its dimer have been computed at the B3LYP/aug-cc-pVTZ level. The theoretical chemical shifts δ(^1^H) in these structures can be divided into three groups. The first includes hydrogen atoms that do not participate in the formation of the H-bond, and their chemical shifts do not exceed 3 ppm. The second group consists of H atoms forming intermolecular C–H∙∙∙O bonds, and their chemical shifts lie within 4–5 ppm. The last group includes hydrogen atoms that are involved in the formation of intramolecular C–H∙∙∙O bonds, δ(^1^H) > 5 ppm. Due to the rapid rotation of the methyl groups around the CN bonds, the 1H NMR spectrum will actually contain only one CH signal of hydrogen atoms forming inter- and intramolecular C–H∙∙∙O bonds. It is reasonable to expect that under favorable conditions for GB diluted in aprotic solvents, the formation of intermolecular C–H∙∙∙O bonds might be detected by ^1^H NMR as a concentration dependence of CH chemical shifts. According to the results of the calculations, the formation of weaker intermolecular C–H∙∙∙O bonds instead of stronger intramolecular bonds should lead to a high-field shift of the signal of the CH proton located by about 5–6 ppm. 

## 4. Materials and Methods

The spatial structure of the hydrates of organic molecules is determined primarily by intermolecular H-bonds, which are of an electrostatic nature. These H-bonds play a structure-directing role in the crystalline hydrates of organic molecules [54], multicomponent crystals of energetic substances [66] and pharmaceutically active ingredients [24,67]. The metric parameters and energies of H-bonds, the IR and Raman spectra of the crystals under consideration are satisfactorily reproduced in the B3LYP/6-31G** approximation [25,50,54]. Explicit allowance for London dispersion interactions has practically no effect on the calculated values of these characteristics in the crystals under consideration [25,68,69]. To validate the results obtained in the B3LYP/6-31G** approximation, we also performed calculations with functionals and basis sets widely used in the periodic and non-periodic calculations of organic molecules and their complexes, as seen below.

### 4.1. Non-Periodic DFT Computations

Non-periodic computations were performed using the Gaussian16 software package [70]. The molecular structures were plotted using GaussView 6.0.16 [71]. We used the B3LYP [72,73] functional with the all-electron Gaussian-type localized orbital basis sets 6-31G**. To check the results of the B3LYP/6-31G** calculations in non-periodic computations, the B3LYP/6-311++G** and wB97XD/aug-cc-pVDZ approximations were also used. B3LYP/6-311++G** is commonly used in sequential hydration calculations of aliphatic amino acid zwitterions, carboxylic acid anions, GB and choline [12,18,31]. Consideration of a newer DFT functional, such as wB97XD [74], was justified by the need to verify the analysis of noncovalent interactions. All calculated complexes correspond to a minimum on the potential energy surface. 

The energy of the intermolecular interaction in the GB dimer was calculated as the sum of the energies of the components minus the energy (BSSE corrected) of the complex. The BSSE corrections were performed using the counterpoise method [75].

The analysis of the electrostatic potential and electron density gradient fields [76] were performed and visualized in Multiwfn 3.8 [77]

### 4.2. Periodic DFT Computations

Computations with all-electron Gaussian-type localized orbital basis 6-31G** were conducted using the CRYSTAL17 package [78]. We employed B3LYP and PBE [79]. The London dispersion interactions were taken into account by introducing the D3 correction with Becke-Jones damping (PBE-D3) developed by Grimme et al. [80]. B3LYP and PBE-D3 are the most popular functionals in periodic (solid-state) DFT calculations of organic crystals [26,81,82,83,84]. These functionals provide a well-founded trade-off between accuracy and computational speed for experimentally observed properties of multicomponent organic crystals [85,86,87]. The space group and unit cell parameter of the considered crystal obtained from the X-ray diffraction experiment [56] were fixed, and the structural relaxations were restricted to the positional parameters of the atoms (AtomOnly). Tolerance on energy controlling the self-consistent field convergence for geometry optimizations and frequencies computations is set to 10^−10^ and 10^−11^ hartree, respectively. The shrinking factor of the reciprocal space net is set to 3. The structures optimized at the B3LYP/6-31G** and PBE-D3/6-31G** levels are found to correspond to the minimum point on the potential energy surface.

### 4.3. Evaluation of the Enthalpy/energy of Intermolecular H-Bonds and Noncovalent Interactions

There are several empirical approaches to estimating the enthalpy/energy of noncovalent interactions in gas and condensed phases; for example, see [49,50]. Rosenberg’s approach [28] was developed for intermolecular H-bonds, see Equation (1). It uses the H∙∙∙O distance, which can be obtained by neutron diffraction or calculated. The accuracy of the enthalpy is determined by the accuracy with which the H∙∙∙O distance is calculated. To obtain reliable enthalpy values, H∙∙∙O distances were calculated in this work using several approximations, that is, different functionals and basis sets. Note that Equation (1) is not intended to estimate the enthalpy of H∙∙∙H interactions; therefore, their energy was calculated by Equation (2). According to [50], these approaches give close values of the enthalpy/energy of moderate H-bonds (<30 kJ/mol).

The Δ*H*_HB_ values of the O–H∙∙∙O and C–H∙∙∙O bonds were estimated using the Rozenberg approach [28]:

−Δ*H*_HB_ [kJ/mol] = 0.134·R(H∙∙∙O)^−3.05^, (1)

where R(H∙∙∙O) is the H∙∙∙O distance (nm). It was obtained as a result of geometry optimization.

The energy of intermolecular H-bonds E_int_ was evaluated according to ref. [29] as:

E_int_ = 0.429·G_b_, (2)

where G_b_ is the positively defined local electronic kinetic energy density at the H∙∙∙O or H∙∙∙H bond critical point [27]. 

The quantum-topological analysis of the non-periodic and periodic electron density was performed using the programs AIMALL [88] and Topond14 [89], respectively. 

## 5. Conclusions

The specific proton donor properties of glycine betaine are due to its ability to form intermolecular C–H∙∙∙O bonds with the oxygen atom of a water molecule or the carboxylate group of a neighboring glycine betaine. Such bonds form the hydrogen atoms of both the methylene and methyl groups of glycine betaine. The enthalpy of intermolecular C–H∙∙∙O bonds can be significantly greater than 10 kJ/mol. The water molecule can form three H-bonds with glycine betaine: either two O–H∙∙∙O bonds as a proton donor and one C–H∙∙∙O bond as a proton acceptor, or vice versa. The C–H∙∙∙O bonds contribute up to 40% of the total entropy of the glycine betaine–water interaction, which is about 45 kJ/mol. 

It is reasonable to expect that under favorable conditions for glycine betaine diluted in aprotic solvents, the formation of intermolecular C–H∙∙∙O bonds might be detected by proton nuclear magnetic resonance as a concentration dependence of CH chemical shifts. According to the results of calculations, the formation of weaker intermolecular C–H∙∙∙O bonds instead of stronger intramolecular bonds should lead to a high-field shift of the signal of the CH proton.

## Figures and Tables

**Table 1:** H∙∙∙O distances R(H∙∙∙O) in GB dimer calculated using B3LYP/6-31G** and wB97XD/aug-cc-pVDZ and intermolecular H-bond enthalpies, computed using Equation (1).

Fragment ^a^	R(H∙∙∙O), Å		–Δ*H*_HB_, kJ/mol	
	B3LYP/6-31G**	wB97XD/aug-cc-pVDZ	B3LYP/6-31G**	wB97XD/aug-cc-pVDZ
C–H36∙∙∙O2	2.435 (139.6)	2.347 (143.2)	10.0	11.1
C–H32∙∙∙O2	2.250 (144.7)	2.386 (141.2)	12.7	10.6
C–H37∙∙∙O2	2.142 (147.5)	2.175 (148.7)	14.7	14.0
C–H19∙∙∙O21	2.142 (147.5)	2.176 (148.7)	14.7	14.0
C–H11∙∙∙O21	2.250 (144.7)	2.386 (141.2)	12.7	10.6
C–H15∙∙∙O21	2.435 (139.6)	2.347 (143.2)	10.0	11.1
**Total enthalpy**			**74.8**	**71.5**

^a^ See Figure 4.

**Table 2:** H∙∙∙O distances R(H∙∙∙O) in GB crystalline monohydrate, calculated using periodic DFT in the B3LYP/6-31G** and PBE-D3/6-31G** approximations, and intermolecular H-bond enthalpies, computed using Equation (1).

Fragment ^a^	R(H∙∙∙O), Å		–Δ*H*_HB_, kJ/mol	
	B3LYP	PBE-D3	B3LYP	PBE-D3
Water–GB interactions				
O–H12∙∙∙O1	1.676	1.656	31.1	32.3
O–H13∙∙∙O2	1.649	1.633	32.7	33.7
O3∙∙∙H11–C	1.989	1.964	18.5	19.2
O3∙∙∙H2–C	2.277	2.258	12.2	12.5
O3∙∙∙H4–C	2.442	2.459	9.9	9.7
O3∙∙∙H8–C	2.481	2.475	9.4	9.5
O3∙∙∙H3–C	2.543	2.528	8.7	8.9
O3∙∙∙H7–C	2.665	2.531	7.6	8.9
O3∙∙∙H9–C	2.671	2.663	7.5	7.6
**Total enthalpy**			**137.6**	**142.3**
GB–GB interactions				
O1∙∙∙H3–C	2.188	2.175	13.8	14.1
O1∙∙∙H5–C	2.522	2.486	8.9	9.4
O1∙∙∙H6–C	2.647	2.641	7.7	7.8
O1∙∙∙H7–C	2.527	2.518	8.9	9.0
O1∙∙∙H9–C	2.275	2.263	12.3	12.5
O1∙∙∙H10–C	2.205	2.201	13.5	13.6
O2∙∙∙H6–C	2.168	2.158	14.2	14.4
O2∙∙∙H8–C	2.630	2.628	7.9	7.9
O2∙∙∙H10–C	2.253	2.228	12.6	13.1
**Total enthalpy**			**99.8**	**101.8**

^a^ see Figure 5. The atomic number used is included in the CDC file 760253.cif of GB crystalline monohydrate [56].

## Data Availability

I/O files are available from the respective author upon reasonable request.

## Supplementary Materials

The Supplementary Materials are available online at https://www.mdpi.com/article/10.3390/ijms241612971/s1.

## References

[1] Ashraf M., Foolad M.R. (2007). Roles of glycine betaine and proline in improving plant abiotic stress resistance. Environ. Exp. Bot..

[2] El Achkar T., Fourmentin S., Greige-Gerges H. (2019). Deep eutectic solvents: An overview on their interactions with water and biochemical compounds. J. Mol. Liq..

[3] Figueroa-Soto C.G., Valenzuela-Soto E.M. (2018). Glycine betaine rather than acting only as an osmolyte also plays a role as regulator in cellular metabolism. Biochimie.

[4] Di Gioacchino M., Bruni F., Ricci M.A. (2020). Aqueous solution of betaine: Hydration and aggregation. J. Mol. Liq..

[5] Ohsawa S., Tokushima T., Okada K. (2021). Hydration of the zwitterionic and protonated forms of glycine betaine probed by soft X-ray emission spectroscopy coupled with chemometrics. J. Phys. Chem. B.

[6] Stasiulewicz M., Panuszko A., Śmiechowski M., Bruździak P., Maszota P., Stangret J. (2021). Effect of urea and glycine betaine on the hydration sphere of model molecules for the surface features of proteins. J. Mol. Liq..

[7] Novak I. (2021). Photoionization of glycine-betaine: A permanent zwitterion. J. Mol. Liq..

[8] Fedotova M.V., Kruchinin S.E. (2017). Hydration and ion-binding of glycine betaine: How they may be involved in the protection of proteins under abiotic stresses. J. Mol. Liq..

[9] Mojtabavi S., Jafari M., Samadi N., Mehrnejad F., Faramarzi M.A. (2021). Insights into the Molecular-Level details of betaine interactions with Laccase under various thermal conditions. J. Mol. Liq..

[10] Jiang L., Zheng K. (2022). Electronic structures of zwitterionic and protonated forms of glycine betaine in water: Insights into solvent effects from ab initio simulations. J. Mol. Liq..

[11] Derbel N., Hernandez B., Pfluger F., Liquier J., Geinguenaud F., Jaidane N., Lakhdar Z.B., Ghomi M. (2007). Vibrational Analysis of Amino Acids and Short Peptides in Hydrated Media. I. L-glycine and L-leucine. J. Phys. Chem. B.

[12] Panuszko A., Śmiechowski M., Stangret J. (2011). Fourier transform infrared spectroscopic and theoretical study of water interactions with glycine and its N-methylated derivatives. J. Chem. Phys..

[13] Kayi H., Kaiser R.I., Head J.D. (2012). A theoretical investigation of the relative stability of hydrated glycine and methylcarbamic acid—From water clusters to interstellar ices. Phys. Chem. Chem. Phys..

[14] de Tudela R.P., Marx D. (2016). Water-induced Zwitterionization of Glycine: Stabilization Mechanism and Spectral Signatures. J. Phys. Chem. Lett..

[15] Fedotova M.V., Kruchinin S.E. (2012). 1D-RISM study of glycine zwitterion hydration and ion-molecular complex formation in aqueous NaCl solutions. J. Mol. Liq..

[16] Rabdano S.O., Donets A.V., Vovk M.A., Michel D., Chizhik V.I. (2015). “Hydration Shells” of CH2 Groups of ω-Amino Acids as Studied by Deuteron NMR Relaxation. J. Phys. Chem. B.

[17] Jorgensen W.L., Gao J. (1986). Monte Carlo simulations of the hydration of ammonium and carboxylate ions. J. Phys. Chem..

[18] Gojło E., Śmiechowski M., Panuszko A., Stangret J. (2009). Hydration of Carboxylate Anions: Infrared Spectroscopy of Aqueous Solutions. J. Phys. Chem. B.

[19] Del Val C., Bondar A.N. (2017). Charged groups at binding interfaces of the PsbO subunit of photosystem II: A combined bioinformatics and simulation study. Biochim. Biophys. Acta Bioenerg..

[20] Levina E.O., Penkov N.V., Rodionova N.N., Tarasov S.A., Barykina D.V., Vener M.V. (2018). Hydration of the Carboxylate Group in Anti-Inflammatory Drugs: ATR-IR and Computational Studies of Aqueous Solution of Sodium Diclofenac. ACS Omega.

[21] Vener M.V., Makhrov D.E., Voronin A.P., Shalafan D.R. (2022). Molecular Dynamics Simulation of Association Processes in Aqueous Solutions of Maleate Salts of Drug-like Compounds: The Role of Counterion. Int. J. Mol. Sci..

[22] Jiang L., Lai L. (2002). C–H∙∙∙O Hydrogen Bonds at Protein-Protein Interfaces. J. Biol. Chem..

[23] McConnell D.B. (2021). Biotin’s Lessons in Drug Design. J. Med. Chem..

[24] Surov A.O., Voronin A.P., Vener M.V., Churakov A.V., Perlovich G.L. (2018). Specific features of supramolecular organisation and hydrogen bonding in proline cocrystals: A case study of fenamates and diclofenac. CrystEngComm.

[25] Voronin A.P., Surov A.O., Churakov A.V., Parashchuk O.D., Rykounov A.A., Vener M.V. (2020). Combined X-ray Crystallographic, IR/Raman Spectroscopic, and Periodic DFT Investigations of New Multicomponent Crystalline Forms of Anthelmintic Drugs: A Case Study of Carbendazim Maleate. Molecules.

[26] Červinka C., Fulem M. (2019). Cohesive properties of the crystalline phases of twenty proteinogenic α-aminoacids from first-principles calculations. Phys. Chem. Chem. Phys..

[27] Bader R.F.W. (1991). A Quantum Theory of Molecular Structure and Its Applications. Chem. Rev..

[28] Rozenberg M., Loewenschuss A., Marcus Y. (2000). An Empirical Correlation Between Stretching Vibration Redshift and Hydrogen Bond Length. Phys. Chem. Chem. Phys..

[29] Mata I., Alkorta I., Espinosa E., Molins E. (2011). Relationships between Interaction Energy, Intermolecular Distance and Electron Density Properties in Hydrogen Bonded Complexes under External Electric Fields. Chem. Phys. Lett..

[30] Vener M.V., Egorova A.N., Churakov A.V., Tsirelson V.G. (2012). Intermolecular Hydrogen Bond Energies in Crystals Evaluated Using Electron Density Properties: DFT Computations with Periodic Boundary Conditions. J. Comput. Chem..

[31] Kaczmarek D.K., Parus A., Łozynski M., Pernak J. (2020). Use of ammonium salts or binary mixtures derived from amino acids, glycine betaine, choline and indole-3-butyric acid as plant regulators. RSC Adv..

[32] Aikens C.M., Gordon M.S. (2006). Incremental Solvation of Nonionized and Zwitterionic Glycine. J. Am. Chem. Soc..

[33] Markham G.D., Bock C.L., Book C.W. (1997). Hydration of the Carboxylate Group: An ab Initio Molecular Orbital Study of Acetate-Water Complexes. Struct. Chem..

[34] Sironi M., Fornilia A., Fornili S.L. (2001). Water interaction with glycine betaine: A hybrid QM/MM molecular dynamics simulation. Phys. Chem. Chem. Phys..

[35] Lee H., Choi J.H., Verma P.K., Cho M. (2015). Spectral Graph Analyses of Water Hydrogen-Bonding Network and Osmolyte Aggregate Structures in Osmolyte−Water Solutions. J. Phys. Chem. B.

[36] Steiner T. (2002). The Hydrogen Bond in the Solid State. Angew. Chem. Int. Ed..

[37] Gillon A.L., Feeder N., Davey R.J., Storey R. (2003). Hydration in Molecular Crystals-A Cambridge Structural Database Analysis. Cryst. Growth Des..

[38] Infantes L., Fabian L., Motherwell W.D.S. (2007). Organic crystal hydrates: What are the important factors for formation. CrystEngComm.

[39] Meot-Ner M. (2005). The Ionic Hydrogen Bond. Chem. Rev..

[40] Legon A.C. (2010). The Halogen Bond: An Interim Perspective. Phys. Chem. Chem. Phys..

[41] Baiz C.R., Błasiak B., Bredenbeck J., Cho M., Choi J.H., Corcelli S.A., Dijkstra A.G., Feng C.J., Garrett-Roe S., Ge N.H. (2020). Vibrational Spectroscopic Map, Vibrational Spectroscopy, and Intermolecular Interaction. Chem. Rev..

[42] Sheiner S. (2012). The Pnicogen Bond: Its Relation to Hydrogen, Halogen, and Other Noncovalent Bonds. Acc. Chem. Res..

[43] Verevkin S.P., Kondratev S.O., Zaitsau D.H., Zherikova K.V., Ludwig R. (2021). Quantification and understanding of non-covalent interactions in molecular and ionic systems: Dispersion interactions and hydrogen bonding analysed by thermodynamic methods. J. Mol. Liq..

[44] Vazdar M., Heyda J., Mason P.E., Tesei G., Allolio C., Lund M., Jungwirth P. (2018). Arginine “Magic”: Guanidinium Like-Charge Ion Pairing from Aqueous Salts to Cell Penetrating Peptides. Acc. Chem. Res..

[45] Shenderovich I. (2019). The Partner Does Matter: The Structure of Heteroaggregates of Acridine Orange in Water. Molecules.

[46] Inagaki T., Aono S., Nakano H., Yamamoto T. (2014). Like-Charge Attraction of Molecular Cations in Water: Subtle Balance between Interionic Interactions and Ionic Solvation Effect. J. Phys. Chem. B.

[47] Kruchinin S.E., Fedotova M.V. (2021). Ion Pairing of the Neurotransmitters Acetylcholine and Glutamate in Aqueous Solutions. J. Phys. Chem. B.

[48] Shishkina A.V., Ksenofontov A.A., Penkov N.V., Vener M.V. (2022). Diclofenac Ion Hydration: Experimental and Theoretical Search for Anion Pairs. Molecules.

[49] Bartashevich E.V., Tsirelson V.G. (2014). Interplay between non-covalent interactions in complexes and crystals with halogen bonds. Russ. Chem. Rev..

[50] Medvedev A.G., Churakov A.V., Navasardyan M.A., Prikhodchenko P.V., Lev O., Vener M.V. (2022). Fast Quantum Approach for Evaluating the Energy of Non-Covalent Interactions in Molecular Crystals: The Case Study of Intermolecular H-Bonds in Crystalline Peroxosolvates. Molecules.

[51] Rukenstein E., Shulgin I.L., Shulgin L.I. (2007). Cooperativity in Ordinary Ice and Breaking of Hydrogen Bonds. J. Phys. Chem. B.

[52] Tskhovrebov A.G., Novikov A.S., Kritchenkov A.S., Khrustalev V.N., Haukka M. (2020). Attractive halogen···halogen interactions in crystal structure of trans-dibromogold(III) complex. Z. Kristallogr Cryst. Mater..

[53] Wiscons R.A., Bellas M.K., Bennion J.C., Matzger A.J. (2018). Detonation Performance of Ten Forms of 5,5′-Dinitro-2H,2H′-3,3′-Bi-1,2,4-Triazole (DNBT). Cryst. Growth Des..

[54] Surov A.O., Vasilev N.A., Churakov A.V., Parashchuk O.D., Artobolevskii S.V., Alatortsev O.A., Makhrov D.E., Vener M.V. (2021). Two Faces of Water in the Formation and Stabilization of Multicomponent Crystals of Zwitterionic Drug-Like Compounds. Symmetry.

[55] Rai S.K., Gunnam A., Beran G.J., Kaduk J.A., Nangia A.K. (2023). Polymorphs, Solvatomorphs, Hydrate, and Perhydrate of Dabrafenib. Cryst. Growth Des..

[56] Johnstone R.D.L., Lennie A.R., Parsons S., Pidcock E., Warren J.E. (2009). Comparison of the effects of pressure on three layered hydrates: A partially successful attempt to predict a high-pressure phase transition. Acta. Crystallogr. B Struct. Sci. Cryst. Eng. Mater..

[57] Filippov O.A., Tsupreva V.N., Epstein L.M., Lledos A., Shubina E.S. (2008). Intermolecular HH Vibrations of Dihydrogen Bonded Complexes H3EH−···HOR in the Low-Frequency Region: Theory and IR Spectra. J. Phys. Chem. A.

[58] Verma P.K., Lee H., Park J.Y., Lim J.H., Maj M., Choi J.H., Kwak K.W., Cho M. (2015). Modulation of the Hydrogen Bonding Structure of Water by Renal Osmolytes. J. Phys. Chem. Lett..

[59] Lundberg P., Dudman N.P., Kuchel P.W., Wilcken D.E. (1995). 1H NMR determination of urinary betaine in patients with premature vascular disease and mild homocysteinemia. Clin. Chem..

[60] Guo J., Koeppe B., Tolstoy P.M. (2011). Trimethylglycine complexes with carboxylic acids and HF: Solvation by a polar aprotic solvent. Phys. Chem. Chem. Phys..

[61] Ilczyszyn M.M., Wierzejewska M., Ciunik Z. (2000). Crystal structure and vibrational spectra of bis(betaine) sulfamate. Phys. Chem. Chem. Phys..

[62] Shenderovich I.G. (2023). Experimentally Established 15N NMR Absolute Shielding Scale for Theoretical Calculations. J. Phys. Chem. A.

[63] Shenderovich I.G., Limbach H.H. (2021). Solid State NMR for Non Experts: An Overview of Simple but General Practical Methods. Solids.

[64] Makulski W., Jackowski K. (2020). 1H, 13C and 29Si magnetic shielding in gaseous and liquid tetramethylsilane. J. Magn. Reson..

[65] Baldridge K.K., Siegel J.S. (1999). Correlation of Empirical δ(TMS) and Absolute NMR Chemical Shifts Predicted by ab Initio Computations. J. Phys. Chem. A.

[66] Hu L., Staples R.J., Shreeve J.M. (2021). Energetic compounds based on a new fused triazolo [4,5-d]pyridazine ring: Nitroimino lights up energetic performance. Chem. Eng. J..

[67] Surov A.O., Voronin A.P., Drozd K.V., Gruzdev M.S., Perlovich G.L., Prashanth J., Balasubramanian S. (2021). Polymorphic forms of antiandrogenic drug nilutamide: Structural and thermodynamic aspects. Phys. Chem. Chem. Phys..

[68] Katsyuba S.A., Vener M.V., Zvereva E.E., Brandenburg J.G. (2017). The role of London dispersion interactions in strong and moderate intermolecular hydrogen bonds in the crystal and in the gas phase. Chem. Phys. Lett..

[69] Vener M.V., Chernyshov I.Y., Rykounov A.A., Filarowski A. (2018). Structural and spectroscopic features of proton hydrates in the crystalline state. Solid-state DFT study on HCl and triflic acid hydrates. Mol. Phys..

[70] Frisch M.J., Trucks G.W., Schlegel H.B., Scuseria G.E., Robb M.A., Cheeseman J.R., Scalmani G., Barone V., Petersson G.A., Nakatsuji H. (2016). Gaussian 16, Revision A.03.

[71] Dennington R., Keith T.A., Millam J.M. (2016). GaussView, Version 6.

[72] Becke A.D. (1993). Density-functional thermochemistry. III. The role of exact exchange. J. Chem. Phys..

[73] Vosko S.H., Wilk L., Nusair M. (1980). Accurate Spin-Dependent Electron Liquid Correlation Energies for Local Spin Density Calculations: A Critical Analysis. Can. J. Phys..

[74] Chaia J.-D., Head-Gordon M. (2008). Long-range corrected hybrid density functionals with damped atom–atom dispersion corrections. Phys. Chem. Chem. Phys..

[75] Boys S.F., Bernardi F. (1970). The calculation of small molecular interactions by the differences of separate total energies. Some procedures with reduced errors. Mol. Phys..

[76] Shishkina A.V., Zhurov V.V., Stash A.I., Vener M.V., Pinkerton A.A., Tsirelson V.G. (2013). Noncovalent Interactions in Crystalline Picolinic Acid N-Oxide: Insights from Experimental and Theoretical Charge Density Analysis. Cryst. Growth Des..

[77] Lu T., Chen F. (2012). Multiwfn: A multifunctional wavefunction analyzer. J. Comp. Chem..

[78] Dovesi R., Erba A., Orlando R., Zicovich-Wilson C.M., Civalleri B., Maschio L., Rérat M., Casassa S., Baima J., Salustro S. (2018). Quantum-mechanical Condensed Matter Simulations with CRYSTAL. Wiley Interdiscip. Rev. Comput. Mol. Sci..

[79] Perdew J.P., Burke K., Ernzerhof M. (1996). Generalized Gradient Approximation Made Simple. Phys. Rev. Lett..

[80] Grimme S., Ehrlich S., Goerigk L. (2011). Effect of the Damping Function in Dispersion Corrected Density Functional Theory. J. Comput. Chem..

[81] Mohaček-Grošev V., Grdadolnik J., Stare J., Hadži D. (2009). Identification of hydrogen bond modes in polarized Raman spectra of single crystals of α-oxalic acid dihydrate. J. Raman Spectrosc..

[82] Cutini M., Civalleri B., Corno M., Orlando R., Brandenburg J.G., Maschio L., Ugliengo P. (2016). Assessment of Different Quantum Mechanical Methods for the Prediction of Structure and Cohesive Energy of Molecular Crystals. J. Chem. Theory Comput..

[83] Vener M.V., Kharlanov O.G., Sosorev A.Y. (2022). High-Mobility Naphthalene Diimide Derivatives Revealed by Raman-Based In Silico Screening. Int. J. Mol. Sci..

[84] Rogers F.J.M., Radhanpura K., Horvat J., Farrant D. (2022). On the use of a volume constraint to account for thermal expansion effects on the low-frequency vibrations of molecular crystals. Phys. Chem. Chem. Phys..

[85] Vener M.V., Churakov A.V., Voronin A.P., Parashchuk O.D., Artobolevskii S.V., Alatortsev O.A., Makhrov D.E., Medvedev A.G., Filarowski A. (2022). Comparison of Proton Acceptor and Proton Donor Properties of H_2_O and H_2_O_2_ in Organic Crystals of Drug-like Compounds: Peroxosolvates vs. Crystallohydrates. Molecules.

[86] Remoto P.J.G., Berzinš K., Fraser-Miller S.J., Korter T.M., Rades T., Rantanen J., Gordon K.C. (2023). Exploring the Solid-State Landscape of Carbamazepine during Dehydration: A Low Frequency Raman Spectroscopy Perspective. Pharmaceutics.

[87] Voronin A.P., Surov A.O., Churakov A.V., Vener M.V. (2023). Supramolecular Organization in Salts of Riluzole with Dihydroxybenzoic Acids—The Key Role of the Mutual Arrangement of OH Groups. Pharmaceutics.

[88] Keith T.A. (2019). AIMAll, Version 19.10.12.

[89] Gatti C. (1999). TOPOND98 User’s Manual.

