# Supplementary material for: Specific Proton-Donor Properties of Glycine Betaine. Metric Parameters and Enthalpy of Noncovalent Interactions in its Dimer, Water Complexes and Crystalline Hydrate
Source: Int J Mol Sci. 2023 Aug 19;24(16):12971. doi: 10.3390/ijms241612971 (PMC10455243; doi:10.3390/ijms241612971)
Supplement: Supplementary file 1 [file ijms-24-12971-s001.zip › ijms-2548358-supplementary.pdf]

Supporting information

Specific proton-donor properties of glycine betaine. Metric parameters and enthalpy of noncovalent interactions in its dimer, water complexes and crystalline hydrate.

Nikita E. Frolov [a)], Anastasia V. Shishkina [b)], Mikhail V. Vener [*c)]

[a)] *V. M. Gorbatov Federal Research Center for Food Systems, Talalikhina St. 26, Moscow 109316, Russia; frolovne24@gmail.com*

[b)] *Northern (Arctic) Federal University, Severnaya Dvina Emb. 17, Arkhangelsk 163001, Russia; a.shishkina@narfu.ru*

[c)] *Kurnakov Institute of General and Inorganic Chemistry, Russian Academy of Sciences, Leninskii prosp. 31, Moscow 119991, Russia; vener@igic.ras.ru*

**Correspondence: vener@igic.ras.ru*;

Table S1. H···O distances *R*(H···O) in global-minimum structures of complexes of GB with water, calculated using the B3LYP/6-31G** level (A). Also shown are the H···O distances calculated by B3LYP/6-311++G** (B) and wB97XD/aug-cc-pVDZ (C) for similar structures. Intermolecular H-bond enthalpies are estimated according to equation 1.

GB•(H_2O)						
Fragment[a)]	*R*(H···O), Å			$-\Delta H_{HB}$, kJ/mol		
	A	B	C	A	B	C
O–H21…O5	1.753	1.757	1.709	27.1	26.9	29.3
C–H12…O20	2.272	2.368	2.246	12.3	10.8	12.7
C–H2…O20	2.288	2.365	2.328	12.1	10.9	11.4
Total enthalpy				51.5	48.6	53.4
GB•(H_2O)$_2$						
Fragment[a)]	*R*(H···O), Å			$-\Delta H_{HB}$, kJ/mol		
	A	B	C	A	B	C
O–H21…O5	1.736	1.740	1.730	27.9	27.8	28.3
O–H25…O19	1.736	1.740	1.712	27.9	27.8	29.2
C–H17…O23	2.218	2.284	2.208	13.2	12.1	13.4
C–H12…O20	2.219	2.282	2.332	13.2	12.1	11.4
C–H3…O23	2.304	2.454	2.364	11.8	9.7	10.9
C–H2…O20	2.303	2.451	2.450	11.8	9.8	9.8
Total enthalpy				105.8	99.3	103.0
GB•(H_2O)$_3$						
Fragment[a)]	*R*(H···O), Å			$-\Delta H_{HB}$, kJ/mol		
	A	B	C	A	B	C
O–H28…O23	1.724	1.760	1.742	28.6	26.8	27.7
O–H22…O26	1.847	1.896	1.906	23.1	21.4	21.0
O–H24…O5	1.978	2.646	2.358	18.8	7.7	11.0
O–H25…O19	1.990	1.804	1.836	18.4	24.9	23.6
O-H21…O5	2.040	2.164	2.021	17.1	14.3	17.6
C–H12…O20	2.282	2.407	2.368	12.1	10.3	10.8
C–H2…O20	2.471	2.588	2.510	9.5	8.3	9.1
C–H12…O26	2.914	3.362	2.862	5.8	3.7	6.1
Total enthalpy				133.4	117.4	126.9
GB•(H_2O)$_4$						
Fragment[a)]	*R*(H···O), Å			$-\Delta H_{HB}$, kJ/mol		
	A	B	C	A	B	C
O–H27…O5	1.789	1.796	1.758	25.5	25.2	26.9
O–H25…O20	1.827	1.951	1.885	23.9	19.6	21.7
O–H22…O29	1.913	2.091	2.049	21.9	15.8	16.9
O–H30…O19	1.881	1.744	1.721	20.8	27.6	28.7
O–H21…O5	1.942	1.909	1.852	19.9	20.9	22.9
O–H24…O19	2.002	3.297	3.141	18.1	4.0	4.6
C–H13…O23	2.126	2.160	2.408	15.1	14.4	10.3
C–H12…O26	2.222	2.340	2.251	13.2	11.2	12.7
C–H2…O26	2.244	2.332	2.337	12.8	11.4	11.3
Total enthalpy				171.2	150.1	156.0
GB•(H_2O)$_5$						

Fragment[a)]	R(H···O), Å			$-\Delta H_{HB}$, kJ/mol		
	A	B	C	A	B	C
O–H25…O20	1.819	1.938	1.891	24.2	20.0	21.5
O–H27…O5	1.812	1.812	1.785	24.5	24.5	25.7
O–H33…O19	1.825	1.803	1.782	24.0	24.9	25.8
O–H22…O29	1.897	2.089	2.060	21.3	15.9	16.6
O–H30…O19	1.868	1.779	1.750	22.4	25.9	27.3
O–H21…O5	1.960	1.901	1.861	19.3	21.2	22.6
O–H24…O19	2.084	3.330	3.192	16.0	3.8	4.4
C–H12…O26	2.210	2.328	2.266	13.4	11.4	12.4
C–H2…O26	2.268	2.415	2.358	12.4	10.2	11.0
C–H3…O32	2.300	2.384	2.383	11.8	10.6	10.6
C–H17…O32	2.281	2.325	2.287	12.2	11.5	12.1
C–H16…O23	2.285	2.232	2.255	12.1	13.0	12.6
C–H13…O23	2.336	2.799	2.409	11.3	6.5	10.3
Total enthalpy				224.9	199.4	212.9

[a)] the numbering of atoms is shown on Figures 1, 2 and 3.

Table S2. H···O distances *R*(H···O) in structures of GB•(H_2O) complexes [a)], calculated using B3LYP/6-31G** and intermolecular H-bond enthalpy/energy, computed using Eqs. (1) and (2).

Local minimum structure GB_H2O_2 *E* = – 478.577416/*– 478.729306*/– **478.498812** a.u.)[b)]			
Fragment	*R*(H···O), Å	$-\Delta H_{HB}$, kJ/mol	E_{int}, kJ/mol
O-H21…O19	1.974	18.9	22.4
O-H22…O5	2.204	13.5	14.8
C-H13…O20	2.180	14.0	14.3
Total enthalpy/energy, kJ/mol		46.4	51.5
Global minimum structure GB_H2O_1 (*E* = – 478.581047/*– 478.730139*/– **478.500944** a.u.)			
Fragment	*R*(H···O), Å	$-\Delta H_{HB}$, kJ/mol	E_{int}, kJ/mol
O-H21…O5	1.753	27.1	32.8
C-H12…O20	2.272	12.3	12.6
C-H2…O20	2.288	12.1	13.3
Total enthalpy/energy, kJ/mol		51.5	58.7
Local minimum structure GB_H2O_3 (*E* = – 478.576123/*– 478.730077*/– **478.501136** a.u.)[a)]			
Fragment	*R*(H···O), Å	$-\Delta H_{HB}$, kJ/mol	E_{int}, kJ/mol
O-H22…O19	1.900	21.2	24.2
C-H17…O20	2.506	9.1	8.9
C-H3…O20	2.794	6.5	5.8
Total enthalpy/energy, kJ/mol		36.8	38.9

[a)] See Figs. 1 and S1.
[b)] Sum of electronic and zero-point energies computed at the B3LYP/6-31G** level. Energies computed at the B3LYP/6-311++G** level are given by script and at the wB97XD/aug-cc-pVDZ level highlighted in bold.

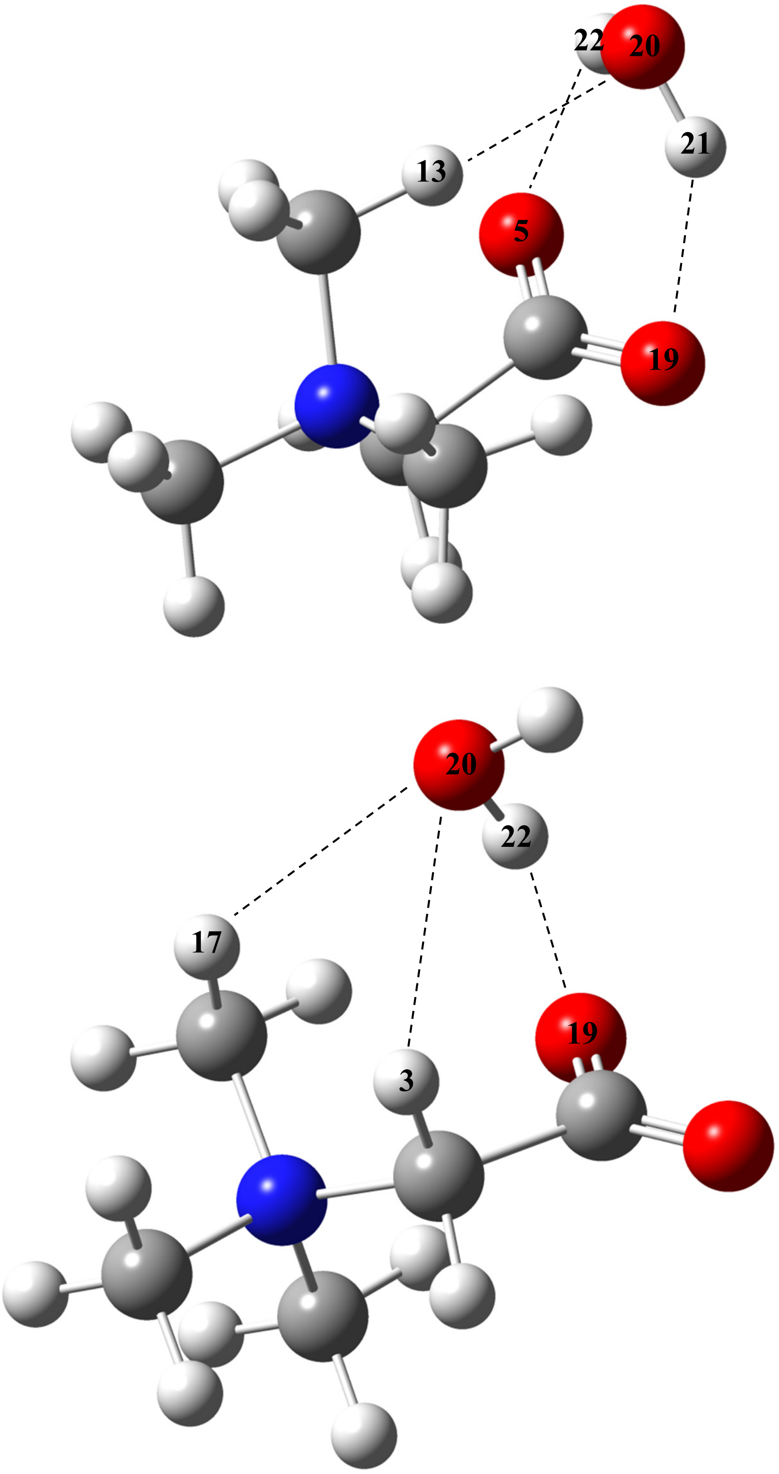

Figure S1. The local-minimum structures GB•(H_2O) complexes: GB_H2O_2 (upper panel) and GB_H2O_3 (lower panel). H-bonds are given by dotted lines.

Table S3. H···O distances R(H···O) in structures of GB•$(H_2O)_2$ complexes [a)], calculated using B3LYP/6-31G** and intermolecular H-bond enthalpy/energy, computed using Eqs. (1) and (2).

Local minimum structure GB_2H2O (*E* = –554.993511/*–555.180032*/–**554.912438** a.u.)[a)]			
Fragment	R(H···O), Å	$-\Delta H_{HB}$, kJ/mol	E_{int}, kJ/mol
O-H21…O5	1.875	22.1	24.2
O-H24…O19	1.944	19.8	21.1
O-H25…O20	2.086	16.0	20.0
Total enthalpy/energy, kJ/mol		57.9	65.3

Global minimum structure GB_2H2O_1 (*E* = -554.999226/*–555.181925*/–**554.917030** a.u.)[a)]			
Fragment	R(H···O), Å	$-\Delta H_{HB}$, kJ/mol	E_{int}, kJ/mol
O-H21…O5	1.736	27.9	34.6
O-H25…O19	1.736	27.9	34.6
C-H17…O23	2.218	13.2	13.8
C-H12…O20	2.219	13.2	13.7
C-H3…O23	2.304	11.8	12.9
C-H2…O20	2.303	11.8	12.9
Total enthalpy/energy, kJ/mol		105.8	122.5

Local minimum structure GB_2H2O_2 (*E* = -554.997421/*–555.178619*/–**554.912893** a.u.)[a)]			
Fragment	R(H···O), Å	$-\Delta H_{HB}$, kJ/mol	E_{int}, kJ/mol
O-H25…O5	1.856	22.8	26.0
O-H21…O5	1.971	19.0	22.0
O-H22…O23	1.985	18.6	21.6
C-H12…O20	2.236	12.9	13.4
C-H2…O20	2.573	8.4	9.4
Total enthalpy/energy, kJ/mol		81.7	92.4

[a)] See Figs. 1 and S2.
[b)] Sum of electronic and zero-point energies computed at the B3LYP/6-31G** level. Energies computed at the B3LYP/6-311++G** level are given by script and at the wB97XD/aug-cc-pVDZ level highlighted in bold.

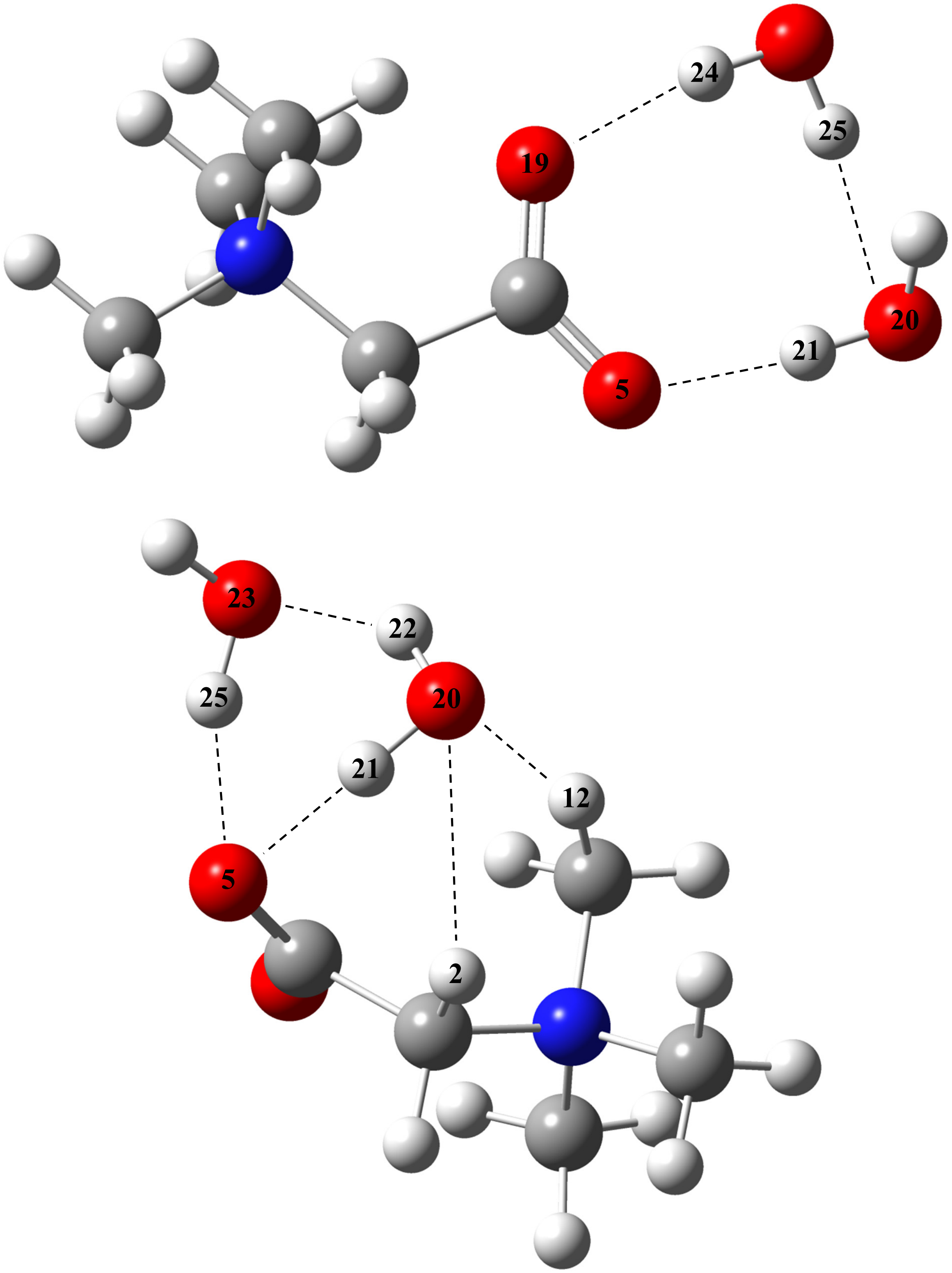

Figure S2. The local-minimum structures GB•(H_2O)$_2$ complexes: GB_2H2O (upper panel) and GB_2H2O_2 (lower panel). H-bonds are given by dotted lines.

Table S4. H···O distances R(H···O) in structures of GB•$(H_2O)_3$ complexes [a)], calculated using B3LYP/6-31G** and intermolecular H-bond enthalpy/energy, computed using Eqs. (1) and (2).

Local minimum structure GB_3H2O (E = –631.419152/*–631.632731*/–**631.329613** a.u.)			
Fragment	R(H···O), Å	$-\Delta H_{HB}$, kJ/mol	E_{int}, kJ/mol
O-H27…O5	1.780	25.9	31.2
O-H21…O5	1.778	26.0	30.8
O-H25…O20	1.898	21.3	25.2
O-H24…O19	1.954	19.5	22.1
C-H13…O23	2.117	15.3	16.3
C-H12…O26	2.233	12.2	13.7
C-H2…O26	2.277	13.0	13.0
Total enthalpy/energy, kJ/mol		133.2	152.3

Global minimum structure GB_3H2O_1 (E = –631.423312/*–631.634229*/–**631.329407** a.u.)[a)]			
Fragment	R(H···O), Å	$-\Delta H_{HB}$, kJ/mol	E_{int}, kJ/mol
O-H28…O23	1.724	28.6	36.0
O-H22…O26	1.847	23.1	27.6
O-H24…O5	1.978	18.8	22.0
O-H25…O19	1.990	18.4	21.6
O-H21…O5	2.040	17.1	18.5
C-H12…O20	2.282	12.1	12.4
C-H2…O20	2.471	9.5	9.8
C-H12…O26	2.914	5.8	4.7
Total enthalpy/energy, kJ/mol		133.4	152.6

Local minimum structure GB_3H2O_2 (E = –631.416654/*–631.632636*/–**631.330693** a.u.)[a)]			
Fragment	R(H···O), Å	$-\Delta H_{HB}$, kJ/mol	E_{int}, kJ/mol
O-H24…O2	1.764	26.6	32.4
O-H27…O3	1.789	25.5	30.6
O-H22…O3	2.091	15.9	17.8
O-H21…O2	2.179	14.0	15.6
C-H13…O23	2.206	13.5	14.1
C-H18…O23	2.269	12.3	13.2
C-H19…O26	2.323	11.9	12.3
C-H11…O26	2.311	11.7	11.7
C-H12…O20	2.298	11.5	11.4
Total enthalpy/energy, kJ/mol		142.9	159.1

Local minimum structure GB_3H2O_3 (E = -631.418208/*–631.631206*/–**631.325752** a.u.)[a)]			
Fragment	R(H···O), Å	$-\Delta H_{HB}$, kJ/mol	E_{int}, kJ/mol
O-H21…O5	1.697	30.0	37.3
O-H24…O26	1.858	23.2	27.3
O-H28…O19	1.846	22.7	26.6
O-H27…O20	1.979	18.7	21.6
C-H13…O23	1.989	18.5	21.2
O-H25…O20	2.040	17.1	18.9
Total enthalpy/energy, kJ/mol		130.2	152.8

[a)] See Figs. 2 and S3.

[b)] Sum of electronic and zero-point energies computed at the B3LYP/6-31G** level. Energies computed at the B3LYP/6-311++G** level are given by script and at the wB97XD/aug-cc-pVDZ level highlighted in bold.

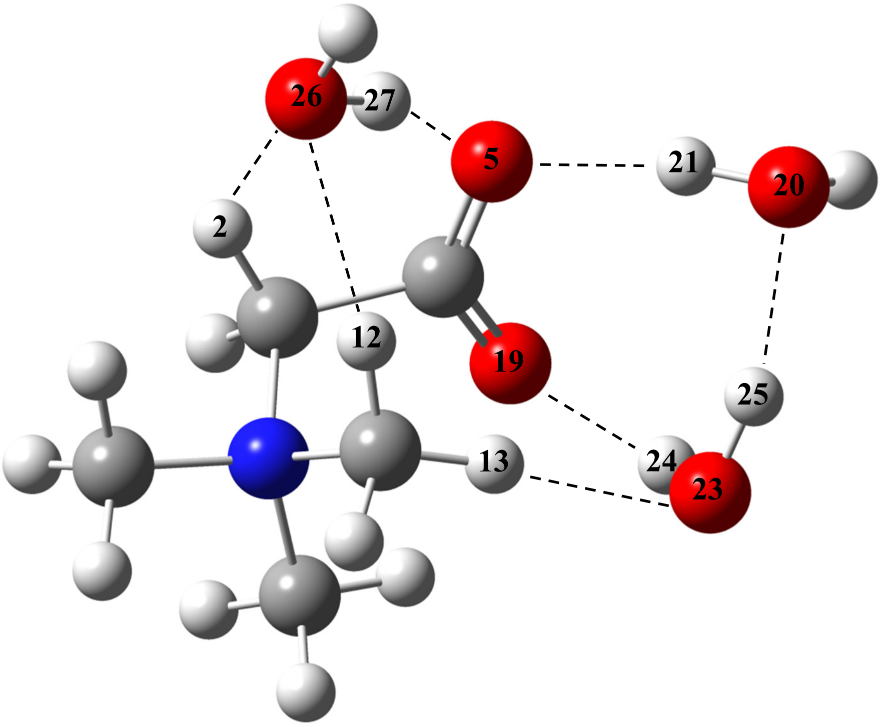
26
27
5
21
20
2
12
19
25
13
24
23

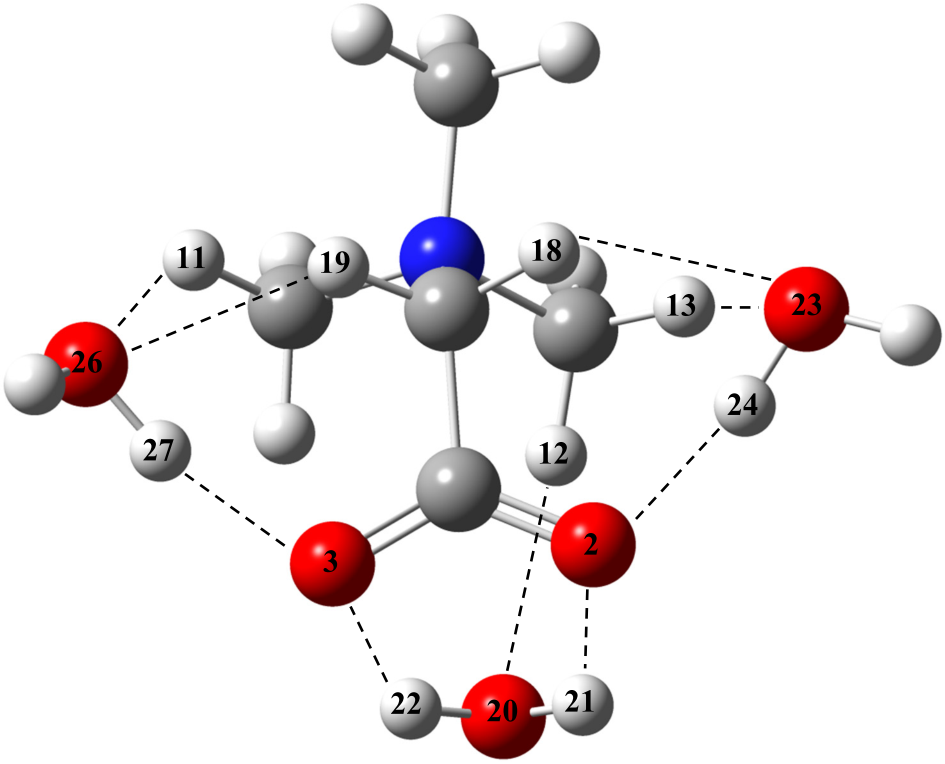
11
19
18
26
13
23
24
27
12
3
2
22
20
21

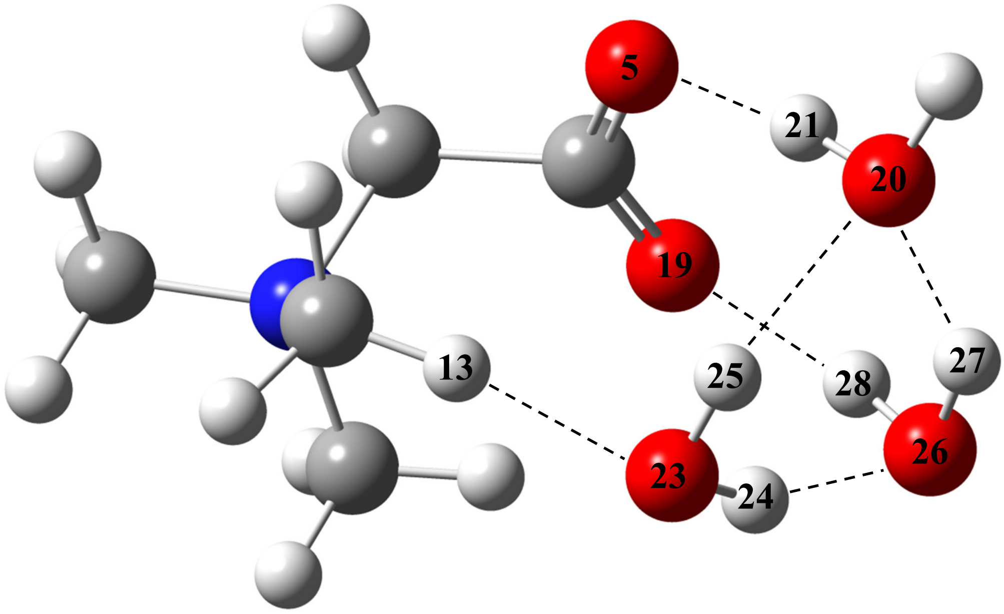

Figure S3. The local-minimum structures GB•$(H_2O)_3$ complexes: GB_3H2O (upper panel), GB_3H2O_2 (middle panel), GB_3H2O_3 (lower panel). H-bonds are given by dotted lines.

Table S5. H···O distances R(H···O) in structures of GB•$(H_2O)_4$ complexes [a)], calculated using B3LYP/6-31G** and intermolecular H-bond enthalpy/energy, computed using Eqs. (1) and (2).

Local minimum structure GB_4H2O (E = –707.835826/*–708.083623*/– **707.744189** a.u.)			
Fragment	R(H···O), Å	$-\Delta H_{HB}$, kJ/mol	E_{int}, kJ/mol
O-H21…O5	1.782	25.8	30.5
O-H27…O5	1.792	25.4	30.3
O-H30…O19	1.807	24.7	29.3
O-H25…O20	1.884	21.8	25.9
O-H24…O19	2.000	18.1	20.3
C-H12…O26	2.214	13.3	14.1
C-H3…O29	2.303	11.8	12.7
C-H2…O26	2.296	11.9	12.3
C-H17…O29	2.284	12.1	12.1
C-H13…O23	2.324	11.5	11.2
C-H16…O23	2.398	10.4	9.4
Total enthalpy/energy, kJ/mol		186.8	208.1

Global minimum structure GB_4H2O_2 (E = –707.836233/*–708.083771*/–**707.742706** a.u.)[a)]			
Fragment	R(H···O), Å	$-\Delta H_{HB}$, kJ/mol	E_{int}, kJ/mol
O-H27…O5	1.789	25.5	30.5
O-H25…O20	1.827	23.9	29.3
O-H22…O29	1.913	21.9	24.5
O-H30…O19	1.881	20.8	24.3
O-H21…O5	1.942	19.9	21.6
O-H24…O19	2.002	18.1	20.1
C-H13…O23	2.126	15.1	16.2
C-H12…O26	2.222	13.2	13.8
C-H2…O26	2.244	12.8	13.8
Total enthalpy/energy, kJ/mol		171.2	194.1

[a)] See Figs. 2 and S4.
[b)] Sum of electronic and zero-point energies computed at the B3LYP/6-31G** level. Energies computed at the B3LYP/6-311++G** level are given by script and at the wB97XD/aug-cc-pVDZ level highlighted in bold.

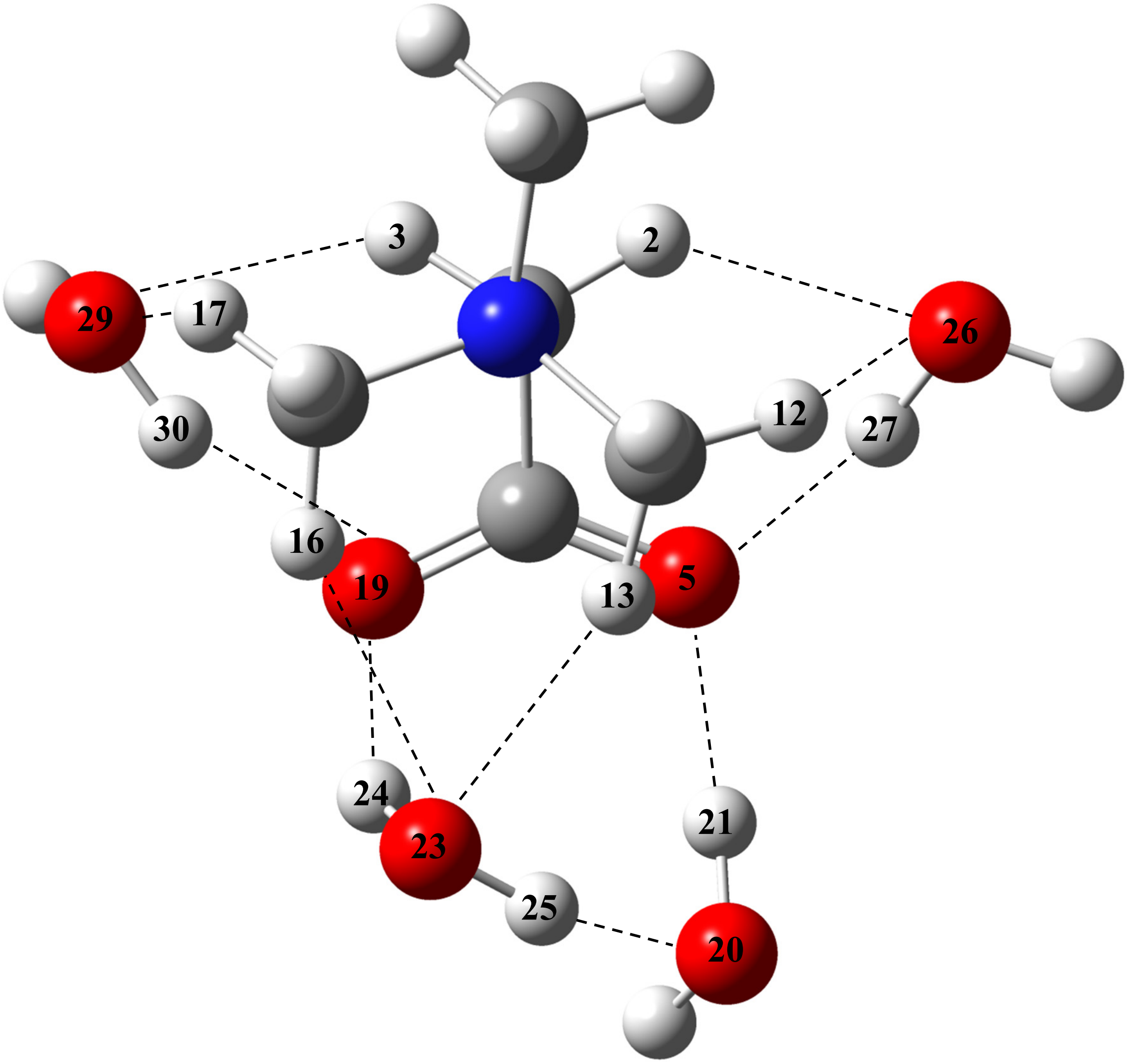

Figure S4. The local-minimum structure GB•$(H_2O)_4$ complex GB_4H2O_2. H-bonds are given by dotted lines.

Table S6. Intermolecular H-bond energies E_{int} in GB dimer evaluated using Eq. (2).

Fragment [a)]	E_{int}, kJ/mol	
	B3LYP/6-31G**	wB97XD/aug-cc-pVDZ
C-H36…O2	8.8	9.9
C-H32…O2	13.1	10.6
C-H37…O2	16.6	14.8
H19-H37	6.9	7.4
C-H19…O21	16.6	14.7
C-H11…O21	13.1	10.6
C-H15…O21	8.8	9.9
Total energy, kJ/mol	83.9	77.9

[a)] See Fig. 4.

Table S7. Intermolecular H-bond energies E_{int}, in GB crystalline hydrate computed using Eq. (2).

Fragment[a)]	E_{int}, kJ/mol	
	B3LYP/6-31G**	PBE-D3/6-31G**
Water-GB interactions		
O-H12…O1	42.0	43.3
O-H13…O2	45.2	45.8
C-H2…O3	13.3	13.5
C-H3…O3	7.8	7.9
C-H4…O3	12.9	12.3
C-H9…O3	7.3	7.3
C-H8…O3	8.7	8.6
C-H7…O3	8.1	8.2
C-H11…O3	22.6	23.1
C-H1…O3	3.4	3.4
Total energy, kJ/mol	171.3	173.4
GB-GB interactions		
C-H10…O1	15.5	15.5
C-H3…O1	17.0	17.1
C-H9…O1	13.1	13.1
C-H5…O1	7.8	8.1
C-H7…O1	9.0	9.2
C-H6…O1	7.2	7.2
C-H6…O2	16.6	16.7
C-H10…O2	15.3	15.8
C-H7…O2	9.3	-
C-H8…O2	6.9	6.9
H2-H5	6.8	6.9
H1-H5	8.6	8.6
H2-H2	3.8	3.8
H4-H9	7.6	7.5
H8-H8	3.8	3.9
H6-H11	7.4	7.4
Total energy, kJ/mol	155.7	147.7

[a)] the numbering of atoms is shown on Figure 5
